# Editorial: The use of plant metabolites to ameliorate sequelae of chemotherapy

**DOI:** 10.3389/fphar.2023.1320139

**Published:** 2023-11-07

**Authors:** Cijo George Vazhappilly, Shoib Sarwar Siddiqui, Ruby John Anto, Rajan Radhakrishnan, Naveen Kumar Devanga Ragupathi

**Affiliations:** ^1^ Department of Biotechnology, School of Arts and Sciences, American University of Ras Al Khaimah, Ras Al-Khaimah, United Arab Emirates; ^2^ School of Life and Medical Sciences, College Lane Campus, University of Hertfordshire, Hatfield, United Kingdom; ^3^ Division of Cancer Research, Rajiv Gandhi Centre for Biotechnology, Thiruvananthapuram, India; ^4^ Molecular Bioassay Laboratory, Institute of Advanced Virology, Thiruvananthapuram, India; ^5^ College of Medicine, Mohammed Bin Rashid University of Medicine and Health Sciences, Dubai, United Arab Emirates; ^6^ Department of Research and Development, Bioberrys Healthcare and Research Centre, Vellore, India; ^7^ Department of Chemical and Biological Engineering, The University of Sheffield, Sheffield, United Kingdom

**Keywords:** cancer, plant metabolites, chemotherapy, flavonoids, DNA damage

Cancer continues to be a fatal disease with a high annual mortality rate, globally. Despite medical advancements, cancer treatments are often associated with significant adverse effects, posing a great challenge to chemotherapy. Chemotherapy is the first-line treatment for several solid and liquid tumors and has effectively increased the patient survival rate. However, most approved chemotherapeutic drugs have relatively low tumor specificity and high toxicity (Schirrmacher, 2019), causing adverse effects that can be mild (grade 1), moderate (grade 2), severe (grade 3), or life-threatening (grade 4). The immediate adverse effects are observed on hair, skin, liver, bone marrow, blood, gastrointestinal tract, and kidneys. Most chemotherapeutic drugs target cellular DNA and/or RNA metabolism through various mechanisms including the production of reactive oxygen species (ROS), thereby affecting cell proliferation and cell cycle progression of cancerous cells (George et al., 2017). Excessive ROS generation may however also impact normal cells and tissues, leading to various toxic effects such as cardiotoxicity, nephrotoxicity, or neurotoxicity (Hu et al., 2019).

Since ancient times, natural products are reservoirs of biologically active compounds and have been widely explored for treating disease conditions and for drug discovery. Many drugs used today are of plant origin, and many more are still under clinical trial. Treatment with antioxidant regimens has proven effective in improving chemotherapy-induced toxicities and has increased the overall survival and quality of life of patients with cancer. Endogenous antioxidant defense enzymes (superoxide dismutase, glutathione peroxidase, and catalase), and nonenzymatic exogenous antioxidants (vitamins, minerals, and plant polyphenols) are known to quench ROS activity and therefore counterbalance oxidative microenvironments and protect normal cells from oxidative stress-induced damage (Siddiqui et al., 2020). Furthermore, intake of certain plant-based compounds has been reported to modulate the gut microbiota, and therefore suggests a possible role for microbiota in the effective and safer outcome of chemotherapeutic treatment (Vazhappilly et al., 2021).

In our Research Topic, we aimed to explore and publish such studies that have used plant metabolites, which were able to potentially reduce the side effects of chemotherapy and/or increase the therapeutic effects of chemo drugs. Kuduvalli et al. used one such approach by combining metformin and epigallocatechin gallate (EGCG) together with temozolomide in a glioma-induced xenograft rat model and was successful in demonstrating its effectiveness as a prospective therapy in glioma patients. This combination of drugs with EGCG helped to inhibit tumor progression and increased the survival rate of rats by 50% (Kuduvalli et al.). Genotoxicity, especially vulnerability to DNA stability leading to DNA damage, is the most common threat along with oxidative stress during cancer treatment. Yadav et al. showed that Piper longum extract could inhibit DNA damage, oxidative stress, hepatotoxicity and neurotoxicity in rats by reducing γ-H2AX and 8-hydroxy-2-deoxyguanosine (8-OHdG) expression levels. Quantification of the extract showed the presence of piperine and piperlongumine along with some flavonoids, which were believed to be the reason for this genoprotective effect (Yadav et al.). In another article, Velayutham et al. showed that a natural benzylisoquinoline alkaloid, stylopine, blocks vascular endothelial growth factor (VEGF)-induced VEGFR- 2 activation in osteosarcoma. *In vitro* analysis using stylopine on human MG-63 osteosarcoma cells showed promising anti-proliferative and anti-migratory effects, that would benefit from integration in nanocarriers to minimize side effects of this natural anticancer compound (Velayutham et al.). Taken together, it can be inferred that plant metabolites may serve as protective agents to minimize the damage to normal cells by chemotherapeutic drugs, as well as by anticancer agents that can enhance the potential of chemotherapeutic drugs. These prospective and innovative findings, if clinically proven, can lead to the use of plant metabolites as potential adjuvants to chemotherapy during cancer treatment.

To conclude, the published studies in this Research Topic highlighted the promising effects of various plant metabolites that can be used along with chemotherapeutic drugs to ameliorate the sequelae of chemotherapy. Furthermore, co-supplementation of plant-based compounds and interaction with microbiota would be beneficial to improve the outcomes of chemotherapy and further investigations are required at this direction. Since there is a need to look for strategies to minimize adverse effects of chemotherapeutic drugs, our Research Topic along with other similar studies, may serve and aid other researchers to work with plant metabolites for developing novel adjuvant treatment strategies that might be able to reduce the adverse effects, and would make a significant impact on cancer chemotherapy.
